# A Mobile App for Prevention of Cardiovascular Disease and Type 2 Diabetes Mellitus: Development and Usability Study

**DOI:** 10.2196/35065

**Published:** 2022-05-10

**Authors:** Vera Helen Buss, Marlien Varnfield, Mark Harris, Margo Barr

**Affiliations:** 1 Australian e-Health Research Centre Commonwealth Scientific and Industrial Research Organisation Herston Australia; 2 Centre for Primary Health Care and Equity University of New South Wales Sydney Australia

**Keywords:** mobile health, behavior change intervention, primary prevention, health promotion, cardiovascular disease, diabetes mellitus, type 2, mobile phone

## Abstract

**Background:**

Cardiovascular disease (CVD) and type 2 diabetes mellitus (T2DM) are posing a huge burden on health care systems worldwide. Mobile apps can deliver behavior change interventions for chronic disease prevention on a large scale, but current evidence for their effectiveness is limited.

**Objective:**

This paper reported on the development and user testing of a mobile app that aims at increasing risk awareness and engaging users in behavior change. It would form part of an intervention for primary prevention of CVD and T2DM.

**Methods:**

The theoretical framework of the app design was based on the Behaviour Change Wheel, combined with the capability, opportunity, and motivation for behavior change system and the behavior change techniques from the Behavior Change Technique Taxonomy (version 1). In addition, evidence from scientific literature has guided the development process. The prototype was tested for user-friendliness via an iterative approach. We conducted semistructured interviews with individuals in the target populations, which included the System Usability Scale. We transcribed and analyzed the interviews using descriptive statistics for the System Usability Scale and thematic analysis to identify app features that improved utility and usability.

**Results:**

The target population was Australians aged ≥45 years. The app included 4 core modules (risk score, goal setting, health measures, and education). In these modules, users learned about their risk for CVD and T2DM; set goals for smoking, alcohol consumption, diet, and physical activity; and tracked them. In total, we included 12 behavior change techniques. We conducted 2 rounds of usability testing, each involving 5 participants. The average age of the participants was 58 (SD 8) years. Totally, 60% (6/10) of the participants owned iPhone Operating System phones, and 40% (4/10) of them owned Android phones. In the first round, we identified a technical issue that prevented 30% (3/10) of the participants from completing the registration process. Among the 70% (7/10) of participants who were able to complete the registration process, 71% (5/7) rated the app above average, based on the System Usability Scale. During the interviews, we identified some issues related to functionality, content, and language and clarity. We used the participants’ feedback to improve these aspects.

**Conclusions:**

We developed the app using behavior change theory and scientific evidence. The user testing allowed us to identify and remove technical errors and integrate additional functions into the app, which the participants had requested. Next, we will evaluate the feasibility of the revised version of the app developed through this design process and usability testing.

## Introduction

### Description of the Behaviors

Cardiovascular disease (CVD) and type 2 diabetes mellitus (T2DM) are 2 widely prevalent chronic conditions [1]. They are highly associated with unhealthy lifestyle, including tobacco smoking, alcohol consumption, poor diet, and physical inactivity. This means that preventative interventions that target these 4 behavioral risk factors can help reduce the risk of developing CVD and T2DM. According to the Global Burden of Disease Study 2019, these 4 factors are among the top behavioral risk factors for the total burden of disease [2]. In 2018, tobacco use contributed the most (8.6%) to the total disability-adjusted life years of Australians, followed by overweight and obesity (8.4%), dietary risk factors (5.4%), high blood pressure (5.1%), and alcohol use (4.5%) [3]. The authors of the report estimated that 38% of the burden of disease measured in the Australian Burden of Disease Study 2018 could have been prevented by reducing or avoiding exposure to the modifiable risk factors that were included in the study [3].

### Overview of Existing Mobile Interventions for Chronic Disease Prevention

Mobile health interventions can be used to address these risk behaviors and help people reduce their risk for CVD and T2DM. Many apps have already been developed for the 4 risk behaviors. In a systematic review from 2020, Milne-Ives et al [4] concluded that there was no strong evidence to show that mobile apps can effectively improve health behaviors or outcomes, because only a few studies demonstrated statistically significantly better results in the intervention compared with the control group [4]. Similar results were reported by Palmer et al [5], Marcolino et al [6], Romeo et al [7], and Lunde et al [8]. We conducted a systematic review of mobile health–based multiple risk factor interventions for the prevention of CVD and T2DM [9]. The review included 3 studies on CVD prevention [10-12] and 6 on T2DM prevention [13-18]. Although the evidence was weak, the findings indicated that at least small to moderate reductions in CVD and T2DM risk can be achieved through mobile health interventions [9]. More recently, Redfern et al [19] published the results of a randomized controlled trial of an app-based intervention for CVD prevention. The intervention aimed at changes in diet, physical activity, smoking, and mental health, but not in alcohol consumption [19]. It showed borderline improvements in risk factors (blood pressure and lipids) and risk-related behaviors (physical activity and eHealth literacy) [19]. None of these interventions targeted CVD and T2DM prevention simultaneously, which is a gap that we aimed to address.

A recurring criticism by authors of systematic reviews is the low quality of evidence for the effectiveness of mobile health interventions [4-9,20-25]. Many have highlighted a lack of rigorous reporting on the theory underlying the intervention and the behavior change techniques included in the app [4,5,8,20-25]. Michie et al [26] explained that the interventions need to be accurately and fully described to subsequently understand which parts of behavior change interventions contributed to outcomes. Carraça et al [21] and Black et al [25] found that effective behavior change techniques vary depending on the mode of delivery, for example, between face-to-face and digital interventions. This means that researchers cannot simply refer to the results of face-to-face behavior change interventions when deciding which behavior change techniques are to be included in the app design.

In addition, Palmer et al [5] found that many studies have focused on individual risk factors. In their systematic review of mobile-based interventions for the prevention of noncommunicable diseases, only 2 of the 71 studies targeted smoking, diet, and physical inactivity, and none targeted all the 4 behaviors (ie, smoking, diet, physical inactivity, and alcohol consumption) [5]. Noble et al [27] conducted a systematic review to investigate which behavioral risk factors were related. They identified that the 4 behaviors often occurred in clusters. Therefore, they argued in favor of multiple behavioral risk interventions [27]. Geller et al [28] highlighted that the behavioral risk factors for chronic conditions are overlapping, which means that successfully addressing these factors will reduce the risk for various chronic diseases.

### Objectives

This study formed part of a larger project in which we aimed to develop and evaluate a mobile app–based intervention for CVD and T2DM risk awareness and prevention. The intervention’s goal is to reduce the risk of both CVD and T2DM by targeting smoking, alcohol consumption, poor diet, and physical inactivity. In this paper, we have described the systematic development and refinement of the mobile app through usability testing.

## Methods

### Methodology

This study followed the methodology developed by Tombor et al [29] for the development of digital behavior change interventions. The methodology combines elements of the United Kingdom’s Medical Research Council guidance [30], Multiphase Optimization Strategy [31], and Behaviour Change Wheel (BCW) [32]. Following these multiphase approaches, the development of the app-based intervention was divided into three phases—preparation, design, and piloting.

### Preparation Phase

#### Step 1: Identify Target Behavior

This app-based intervention focused on 4 behaviors: smoking cessation, moderate alcohol intake, healthy diet (ie, adequate fruit and vegetable intake and avoidance of sugary drinks), and physical activity (ie, walking and other forms of exercise). These 4 behaviors are associated with decreased risk of CVD and T2DM [2]. Regarding smoking, the Australian National Strategy aims to prevent the uptake of smoking, encourage smokers to stop as soon as possible, and support former smokers to stay smoke-free [33]. Regarding alcohol, the Australian guidelines recommend ≤10 standard drinks per week and a maximum of 4 drinks on any day [34]. The Australian recommendations for a healthy diet include a combination of 5 serves of vegetables and 2 serves of fruits per day and no sugary drinks [35]. The advice on exercise states at least 150 minutes of physical activity for people aged 45 to 64 years per week and at least 30 minutes on most, if not all, days for people aged ≥65 years [36]. We assumed that not everyone had to change all 4 risk behaviors. The specific target would depend on the individuals and their needs. Long-term behavior changes were required to lower CVD or T2DM risk [37-40].

#### Step 2: Identify the Theoretical Base

We used BCW as a theoretical base and combined it with the Behavior Change Technique Taxonomy (version 1) by Michie et al [41] to develop the app. Michie et al [32] developed BCW based on findings from a systematic review of existing frameworks of behavior change interventions. The researchers assessed the identified frameworks and addressed each of their limitations in a unified framework, namely the BCW [32]. Then, they tested the reliability with which the framework can be applied in practice [32]. BCW incorporates the capability, opportunity, and motivation for behavior change (COM-B) system [32], which describes the 3 components, capability, opportunity, and motivation, that jointly influence behavior. BCW consists of 3 layers that interact with each other [32]. The COM-B system builds the inner layer [32]. Capability describes physical and psychological factors that allow an individual to act on certain behavior [32]. Opportunity consists of physical and social factors that enable behavior [32]. Motivation comprises automatic or reflective thought processes that influence an individual’s action [32]. The next layer consists of 9 intervention functions (education, persuasion, incentivization, coercion, training, enablement, modeling, environmental restructuring, and restrictions), followed by a layer of 9 policy categories (environmental and social planning, communication and marketing, legislation, service provision, regulation, fiscal measures, and guidelines) [32].

#### Step 3: Review Relevant Scientific Literature

We conducted a systematic literature review to assess the current evidence for the effectiveness of mobile health–based interventions in reducing the risk for CVD and T2DM, with a focus on multiple behavioral risk factor interventions [9]. In addition, we conducted a scoping review between August 2019 and August 2020 to identify relevant papers on behavior change, user engagement, and persuasion in the context of digital health, CVD and T2DM risk prediction, goal setting theory, and risk communication.

#### Step 4: Conduct Needs Assessment

The focus of the app-based intervention is the prevention of CVD and T2DM in Australian adults aged ≥45 years through behavior change. The Australian CVD risk guidelines defined CVD “collectively...as coronary heart disease, stroke and other vascular disease including peripheral arterial disease and renovascular disease” [42]. Diagnostic criteria depended on the specific condition. The Royal Australian Commission of General Practitioners defined T2DM as a "chronic and progressive medical condition that results from two major metabolic dysfunctions: insulin resistance and then pancreatic islet cell dysfunction causing a relative insulin deficiency" [43]. The diagnostic criteria comprised presentation of hyperglycemic crisis, a single elevated fasting blood glucose level ≥7 mmol/L, a single hemoglobin A_1c_ ≥6.5%, or a random blood glucose level ≥11.1 mmol/L (the criteria is slightly different for asymptomatic individuals) [43]. We chose the age group for the intervention based on the advice of the Royal Australian Commission of General Practitioners for general practitioners (GPs) to conduct screening for risk factors and potentially initiate preventative measures in the healthy population ≥45 years [44]. For quantitative needs assessment, we reviewed the data from the Australian Burden of Disease Study 2015 [45] and the Australian National Health Survey 2017-18 [46]; these are summarized in Table S1 of Multimedia Appendix 1 [21,23-25,45-55]. For qualitative needs assessment, we studied the audio and video presentations of people’s real-life experiences of aging in Australia by Healthtalk Australia [56].

### Design Phase

#### Step 5: Select Mode of Delivery

We will deliver the intervention via a mobile app. According to the National Health Survey 2017-18 [46], 89.7% of Australians aged ≥45 years stated that they owned a mobile phone or smartphone.

#### Step 6: Select Intervention Components

We selected BCW intervention functions following the affordability, practicability, effectiveness and cost-effectiveness, acceptability, side effects and safety, and equity (APEASE) criteria [57]. The criteria include whether the intervention is within an acceptable budget, whether it can be delivered as designed, whether it delivers desirable outcomes in practice, whether the benefit-cost ratio is favorable, whether relevant stakeholders consider it as appropriate, whether the risk-benefit ratio is favorable, and whether it narrows or widens disparities between different societal groups [57]. We selected 4 intervention functions: education (ie, “increasing knowledge or understanding”), persuasion (ie, “using communication to induce positive or negative feelings or stimulate action”), incentivization (ie, “creating an expectation of reward”), and enablement (ie, “increasing means/reducing barriers to increase capability [beyond education and training] or opportunity [beyond environmental restructuring]”) from the BCW [57]. Furthermore, we picked 2 policy categories: communication and marketing, and service provision. We chose the intervention components based on previous experience with other apps and published literature.

#### Step 7: Specify the Intervention Content by Behavior Change Techniques

We used the Behavior Change Technique Taxonomy (version 1) by Michie et al [41] to select suitable behavior change techniques and connected them to the appropriate intervention functions, COM-B system components, and policy categories. We identified literature that reported on effective behavior change techniques in mobile health interventions. Subsequently, we developed an intervention strategy based on the selected behavior change techniques. Several systematic reviews and meta-analyses as well as other studies have been published, aiming to identify effective behavior change techniques for mobile health interventions, such as the studies by Carraça et al [21], Qin et al [24], Van Rhoon et al [47], Kaner et al [23], Garnett et al [48], McCrabb et al [49], Black et al [25], Schroé et al [54], and Asbjørnsen et al [55] (for an overview of the results, refer to Table S2 in Multimedia Appendix 1). This shows that, currently, there is no absolute answer as to which techniques are effective in practice; however, there is a clear indication for the effectiveness of self-regulatory strategies. Owing to this uncertainty, we could not simply draw on the results from such meta-analyses to select effective behavior change techniques for our intervention.

#### Step 8: Translate the Intervention Into App Features

In regular meetings, the research team and software engineers discussed the practical translation of the intervention into app features, focusing on user-friendliness and accessibility aspects.

#### Step 9: Design a Prototype App

We based the design of the prototype app on previous apps developed by members of the research team [58-60]. These apps have been validated by different stakeholders, including patients and clinicians. With a focus on the APEASE criteria [57], we set the goal of keeping the app design simple and user-friendly and using less internet data volume and less storage space on the smartphone. In addition, the software engineers developed the app such that it was compatible with iPhone Operating System (iOS) and Android systems. The prototype included 4 core modules: risk score, goal setting, health measures, and education.

### Pilot Phase—Step 10: Conduct User Testing

In the next step, we tested the usability of the app iteratively. We anticipated requiring 2 to 3 cycles to remove all the major design issues. Each cycle consisted of 5 participants from the target population (aged ≥45 years, residing in Australia, fluent in written and spoken English, and owning a smartphone with internet access). We based the sample size calculation on previous studies [61-63]. We recruited participants through the institutional Twitter account and by contacting community groups (eg, community choirs, community gardens, and advocacy groups for older Australians). We offered participants a gift voucher worth Aus $20 (US $14) to thank them for their participation. After providing consent, participants received the study instructions, a link to download the app, a dummy profile, and the user guide (Multimedia Appendix 2) via email. The app was available in a test version only; therefore, the iOS users were required to download the TestFlight app first. Once the app was installed, we asked the participants to use the information provided in the dummy profile to register with the app. We did not collect any app data and asked participants to use the dummy information because, at this stage, we were interested only in the user-friendliness of the app. We invited participants to explore the app further and to book a time for a feedback interview. A researcher (VHB) conducted the semistructured phone interviews (refer to Multimedia Appendix 3 for the interview guide). The interviews consisted of questions about the downloading and registration processes and the System Usability Scale [64]. Then, VHB transcribed the interviews verbatim, analyzed the results of the System Usability Scale using descriptive statistics, and conducted a thematic analysis using NVivo (version 12; QSR International). As described by Neubeck et al [65], we classified the findings into three themes: functionality, content, or language and clarity. On the basis of the findings of the thematic analysis, we resolved the identified issues and added features to the app and user guide according to the participants’ feedback. We repeated the steps until we achieved an adequate version of the app that we could use in a feasibility study.

### Ethics Approval

We received ethics approval from the University of New South Wales Australia Human Research Ethics Advisory Panel (approval number HC200069) and reciprocal approval from the Commonwealth Scientific and Industrial Research Organisation Health and Medical Human Research Ethics Committee (approval number 2020_041_RR).

## Results

### Registration Process and General App Features

**Figure 1:**
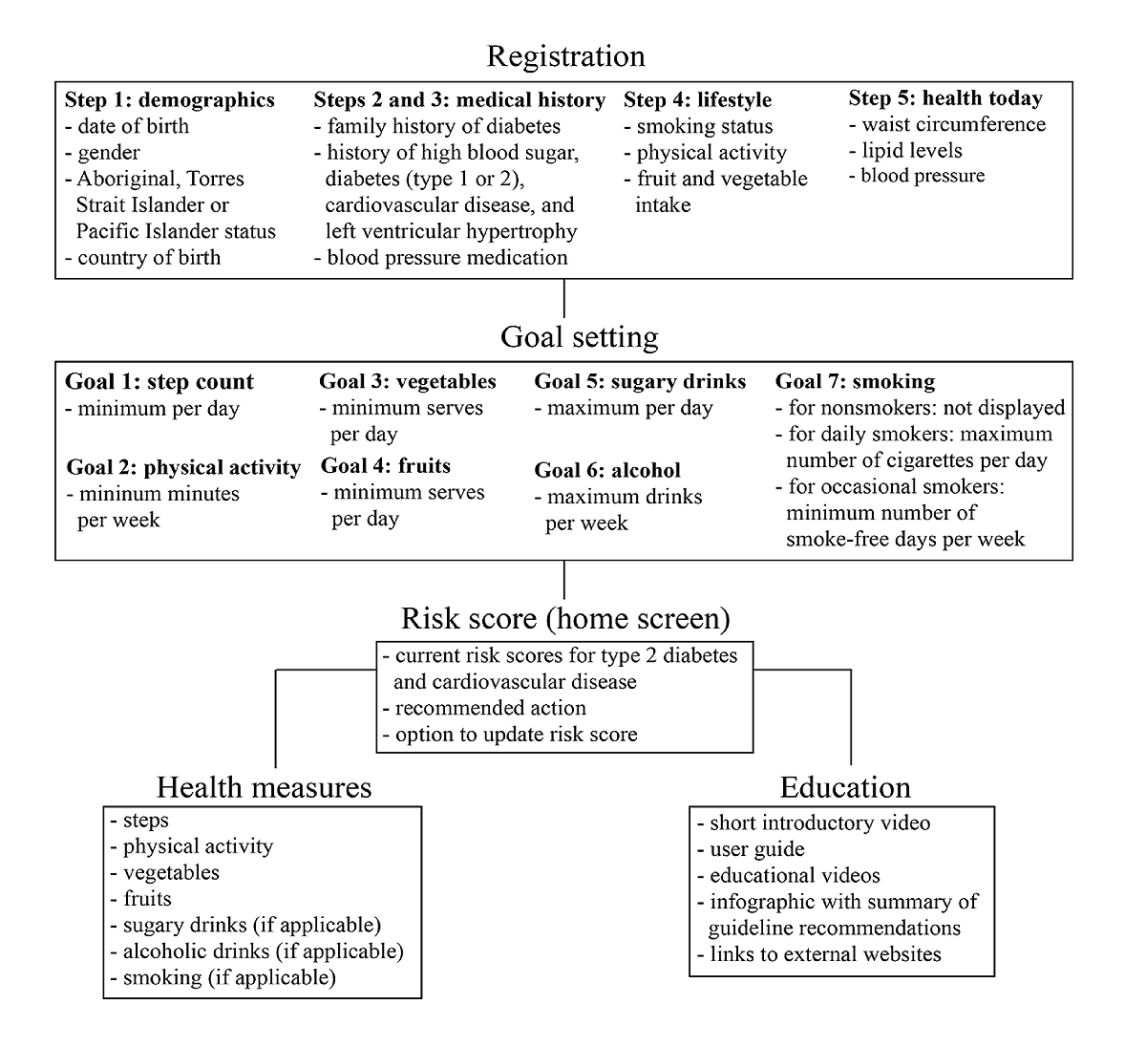
Flowchart of the app, starting from registration.

**Figure 2:**
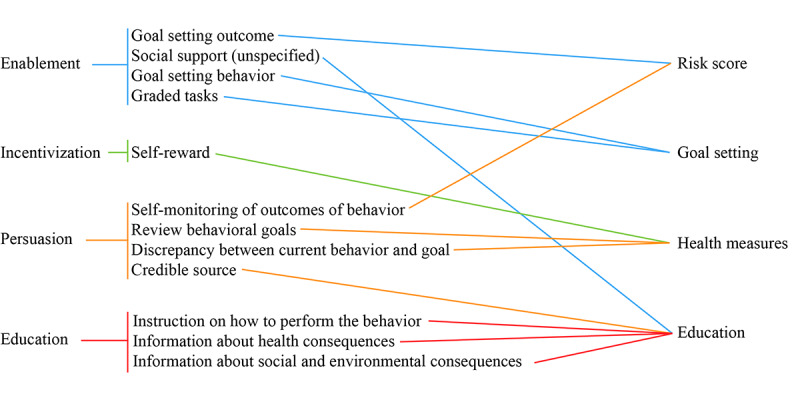
Connections between the selected intervention functions and the 4 app modules via the corresponding behavior change techniques.

The app included the following modules: registration, privacy policy and copyright, risk score, goal setting, health measures, and education. The first 2 modules were general app features, whereas the other 4 modules built the core intervention features. Figure 1 shows the flow of the app, starting from registration. Textbox 1 outlines the principles generated from the COM-B model, and Table 1 shows the connection between the intervention modules and the selected intervention functions, the COM-B system components, and the intervention strategy for the app. It connects the 4 selected intervention functions (education, persuasion, incentivization, and enablement) with the specific behavior change techniques that we selected to achieve behavior change and the corresponding intervention strategies. Figure 2 further outlines the connections between the selected intervention functions and the 4 app modules via the corresponding behavior change techniques. Refer to Table S3 in Multimedia Appendix 1 for more information on the design principles.

**Principles generated from the capability, opportunity, and motivation for behavior change model.**

**Psychological capability**

- Educational videos and links to external websites with evidence-based health information to impart knowledge and train behavioral skills.
- Gradually making behavioral goals more difficult to train the behavioral skills.
- Providing values for goal setting and displaying last achieved values to train the cognitive skills.
- Advice on actions based on personal risks for cardiovascular disease (CVD) and type 2 diabetes mellitus (T2DM) as part of the visualization to improve understanding of adequate measures to achieve target behavior.

**Physical capability**

- Links to websites that provide healthy recipes and exercise instructions to develop skills in cooking and physical activity.

**Social opportunity**

- Advise for contacting general practitioner (included in risk score–related actions, educational videos, and advice if blood pressure or lipid levels not known) for social support.
- Links to websites including support programs (eg, for smoking cessation) for social support.

**Physical opportunity**

- Advise for contacting general practitioner (included in risk score–related actions, educational videos, and advice if blood pressure or lipid levels not known) who can check blood pressure levels and lipid levels or provide pharmacotherapy (eg, for hypertension or dyslipidemia) among others.

**Automatic motivation**

- Visualization of risk for CVD and T2DM displayed on the home screen of the app and the potential to change the risk based on health measures to elicit impulses and counterimpulses related to the target behavior.
- Announcing that the goals have been achieved in 3 consecutive weeks to trigger positive feelings about the behavioral goals.

**Reflective motivation**

- Facilitating self-monitoring of behavioral risk factors and reviewing the progress toward the self-set goals to increase understanding of own behavior and elicit positive or negative feelings about the behavioral goals.
- Highlighting the discrepancy between current behavior and goals to elicit positive or negative feelings and increase understanding of own capabilities about the behavioral goals.
- Encouraging self-reward after achieving weekly goals to elicit positive feelings about the behavioral goals.
- Providing feedback on personal risk of CVD and T2DM in the form of visualization to improve knowledge about own health and elicit positive feelings about the behavioral goals.
- Providing links to websites from credible sources and educational videos to increase knowledge and understanding about the target behavior.
- Setting goals to commit to target behavior and elicit positive feelings about it.

**Table 1:** Core intervention modules with corresponding intervention functions, COM-B^a^ system components, selected behavior change techniques, and intervention strategies.

Module	Linked intervention functions	Key COM-B system components served by module	Selected behavior change techniques	Intervention strategies
Risk score	Enablement and persuasion	Automatic and reflective motivation	Self-monitoring of outcomes of behavior and goal setting (outcome)	Risk score visualization on home screen; outcome goal: low to moderate risk of CVD^b^ and T2DM^c^; and advice on actions based on personal risk, for example, advice to contact their general practitioner
Goal setting	Enablement	Psychological capability and reflective motivation	Goal setting (behavior) and graded tasks	Set behavioral goals for numbers of cigarettes, alcoholic drinks, fruit serves, vegetable serves, and sugary drinks, step count, and minutes of physical activity per day or week based on provided value ranges and advice to gradually make behavioral goals more difficult when they have been achieved in 3 consecutive weeks
Health measures	Persuasion and incentivization	Automatic and reflective motivation	Review behavioral goals, discrepancy between current behavior and goal, and self-reward	Icons and charts showing progress toward self-set daily or weekly goals, display discrepancies between current behavior and previously set goals through color-coding (red circle for negative counting or green circle for positive counting), display last achieved goals, encourage to reward themselves with an object or activity after they achieved their self-set goals, and congratulate when goals were achieved in 3 consecutive weeks
Education	Education, persuasion, and enablement	Physical and psychological capability	Instruction on how to perform the behavior, information about health consequences, information about social and environmental consequences, credible sources, and social support (unspecified)	Links to websites with health information and information about social, environmental, and emotional consequences; short videos on CVD, T2DM, and risk factors with advice to contact general practitioner; all information from credible sources (evidence-based); and links to support groups

^a^COM-B: capability, opportunity, and motivation for behavior change.

^b^CVD: cardiovascular disease.

^c^T2DM: type 2 diabetes mellitus.

### Risk Score

**Figure 3:**
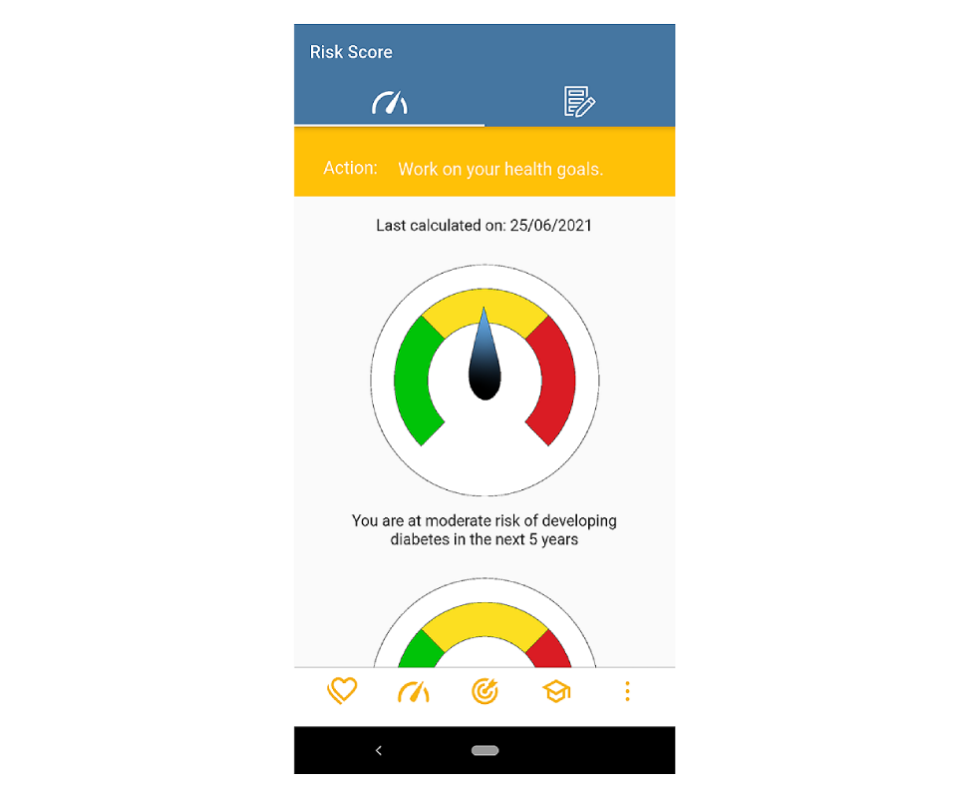
Screenshot of the risk score module.

The long-term goal of the app-based intervention is primary prevention of CVD and T2DM. Therefore, risk presentations for both conditions built a central feature of the app (Figure 3). The risk scores that were embedded into the app were the 1991 version of the Framingham CVD risk score [66] and the Australian Type 2 Diabetes Risk Assessment Tool [67]. These are the standards currently used by clinicians in Australia and endorsed by the Royal Australian Commission of General Practitioners [43,44]. Users provided the information required for the risk calculation during the registration process. We designed the app such that the participants had the option to use Australian averages for lipids and blood pressure based on their age and sex if they did not know their values. Then, they received a recommendation to check the values with their GP. After completing the registration process, users saw their current risk of CVD and T2DM for the next 5 years.

Each time the users opened the app, they saw the risk score screen first. We hypothesized that this would create the impulse to work on the behavioral goals to see a low or moderate disease risk displayed. We followed the principles that we identified during the risk communication scoping review [68-78]: keep the information simple and compact, use absolute instead of relative risk values, combine visuals with text, include information on action to take, and integrate a color scheme that is associated with different risk levels. In particular, the study by Reading Turchioe et al [71] influenced the final version of the visualization. The risk was stratified into 3 different levels (low, moderate, or high) and 3 corresponding actions (“Keep going. You are doing well”; “Work on your health goals”; or “Talk to your GP about your risk”). The recommended action focused on the higher of the 2 risks. Absolute risk values were not displayed because of the difficulty for users to interpret them. Users saw the date on which they had last updated their risk score and can update it at any time. Variables that do not change, such as gender and date of birth, were stored. Physical activity levels were collected through the health measures module. Users could update all other variables in the risk score module.

### Goal Setting

**Figure 4:**
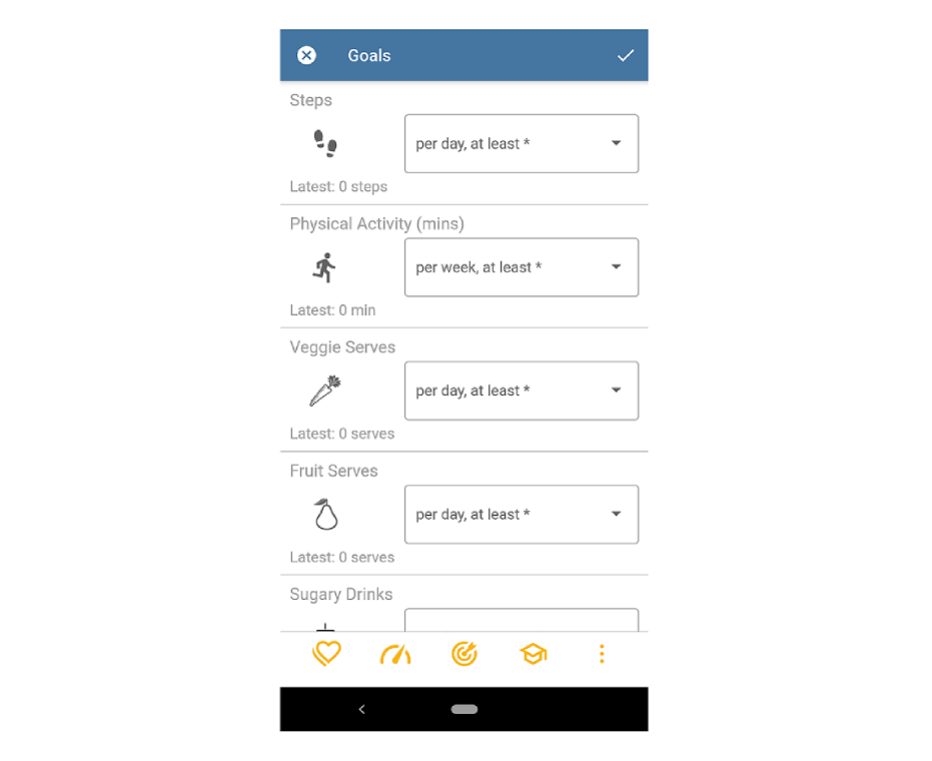
Screenshot of the goal setting module.

The app incorporated a goal setting function (Figure 4), which included proximal, specific goals related to the 4 behavioral factors, smoking, alcohol intake, diet, and physical activity. We designed this module based on the following findings from our literature review, particularly, the principles from the Goal Setting Theory by Locke and Latham [79]. Miller et al [80] stated that combining distal goals with proximal goals is particularly effective because proximal goals are perceived as an important step to achieving personally important distal goals. Locke and Latham [79] explained that specific goals increase self-efficacy and improve performance. Hence, the app incorporated a goal setting function that included proximal, specific goals related to the 4 behavioral factors. The motivation to change behavior is intended to arise from the goal to reduce the risks of CVD and T2DM, which is both a distal goal [79] and the behavior outcome of the intervention. Users had to self-set their behavioral goals, which, according to Locke and Latham [79], increases their self-efficacy compared with assigned goals. There is no goal related to body weight as the studies by Nothwehr and Yang [81] and Shilts et al [82] have shown that more specific goals such as diet and physical activity lead to better results than body weight. Regarding diet-related behavior change, Atkins and Michie [83] outlined that focusing on a specific diet-related target behavior was more effective than focusing on the whole diet. In a study by Rohde et al [84], when asked to rate different food categories, participants voted fruits, vegetables, and sugary drinks as “easy-to-track.” Although whole grains play a significant role in a healthy diet, Foster et al [85] showed in an Australian survey that the general population had poor understanding of the subject. Hence, we assumed that comprehensive education on the subject would be required if it was to be incorporated into the app. The decision to track only sugary drink consumption and not discretionary food consumption was based on findings from 2 studies. Sui et al [86] reported that consumption of discretionary beverages has stronger correlation with high BMI in Australian adults than discretionary foods. Furthermore, Grieger et al [87] showed that raising fruit and vegetable intake can reduce discretionary food intake by subsidizing one for the other. Guided by these findings, we selected fruits, vegetables, and sugary drinks as the diet-related target behaviors.

To support users in their goal setting, the app displayed the last tracked values and offered a range of values for each goal to select from (eg, for vegetables, between 1-5 serves per day). The former helped people to set achievable goals, whereas the latter was consistent with the Australian guidelines. Further support for goal setting could be found in the educational module. To ensure that every user set their goals at least once, they were directed to the goal setting module directly after completing the registration process. Regarding physical activity, users set 2 goals—step count and exercise. This provided users with both daily and weekly goals. Gouveia et al [88] have shown with physical activity that by using a default setting, people may keep this as their goal, even if they could achieve a more ambitious goal. In contrast, not everyone may be able to achieve the guideline recommendations, as argued by Kwasnicka et al [89]. Therefore, users could choose from a wide range of values that allowed for personalized and flexible goals, which Kwasnicka et al [89] argued to be important features in physical activity promotion. In the app, users could select that they did not drink any sugary or alcoholic drinks, which would automatically exclude these from the tracking function. Otherwise, they could select a value from the provided range. The smoking feature was personalized according to the smoking status that the users specified during the registration process.

### Health Measures

**Figure 5:**
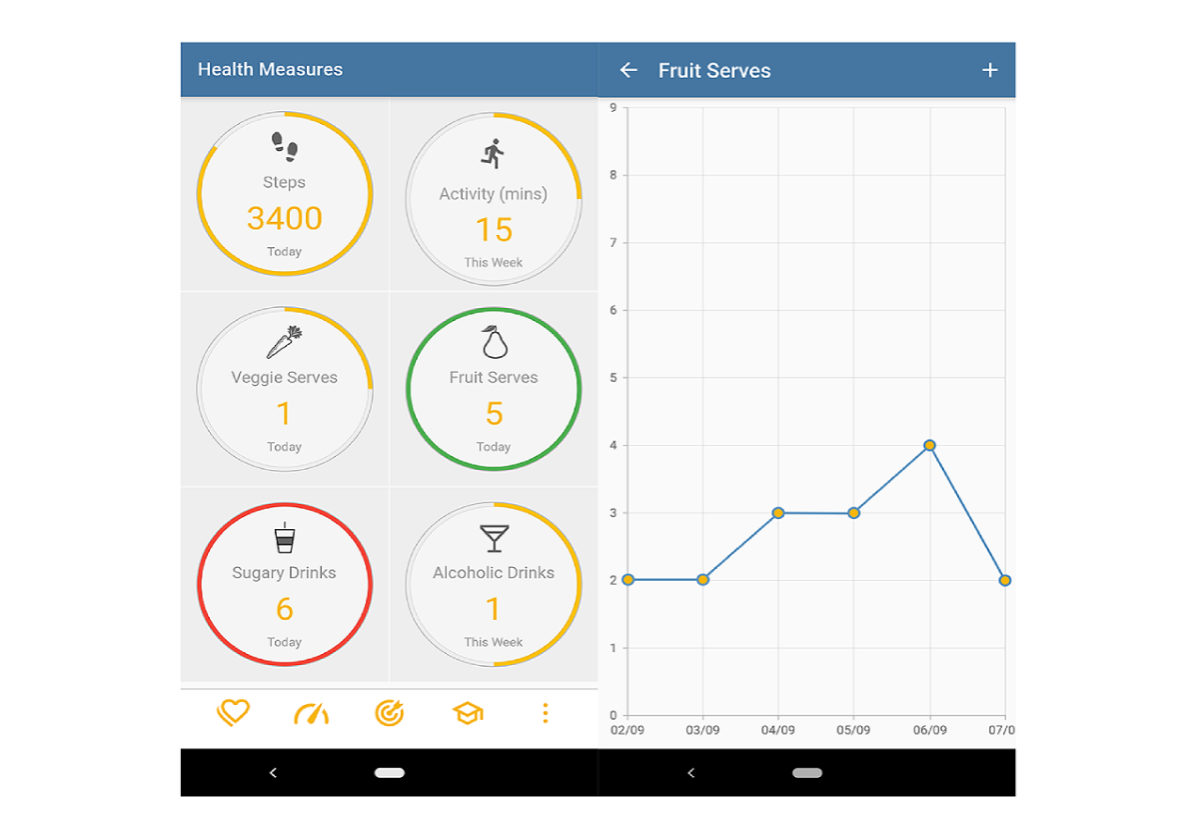
Screenshots of health measures module.

In the health measures module (Figure 5), users could track their behavior and receive feedback on their progress toward the self-set goals. Locke and Latham [79] stated that the combination of single goal feedback with summary feedback has shown to be more effective than one of them alone. In the context of the app, the summary feedback (ie, feedback on the behavior outcome) was in the form of risk scores (ie, risk of CVD and T2DM), whereas the single goal feedback (ie, feedback on the four behaviors: smoking, alcohol, diet, and physical activity) was displayed in the health measures module. Each tracked goal was symbolized by an icon surrounded by a circle. The circle showed the progress. With each step that brought the user closer to their goal, a part of the circle turned yellow. A fully colored circle indicated that the self-set goal had been achieved. For *positive* behaviors (eg, serves of fruits), the circle turned green, and for *negative* behaviors (eg, sugary drinks), the circle turned red. Two types of in-app push notifications encouraged self-reward and graded tasks (ie, increased the difficulty of the goal over time). First, when users achieved their weekly goals, they received a message encouraging them to reward themselves for their success (“You achieved your weekly goals, well done. Think of a way how you can reward yourself for that.”). The message addressed the incentivization intervention function and aimed to motivate users and increase their self-regulation, as postulated by Locke and Latham [79]. Second, when the users achieved the goals in 3 consecutive weeks, a message would pop up to encourage them to set a more ambitious goal (“You achieved your weekly goals 3 times in a row, well done. It’s time to set a more challenging goal.”). This addressed the enablement intervention function and was based on 2 principles. First, Locke and Latham [79] advocated for difficult but attainable goals. Second, Gordon et al [90] found that success in the first week of trying to achieve a new goal is highly determinant of the overall success toward this goal. Graphs showed users their behavior over time (Figure 5).

### Education

**Figure 6:**
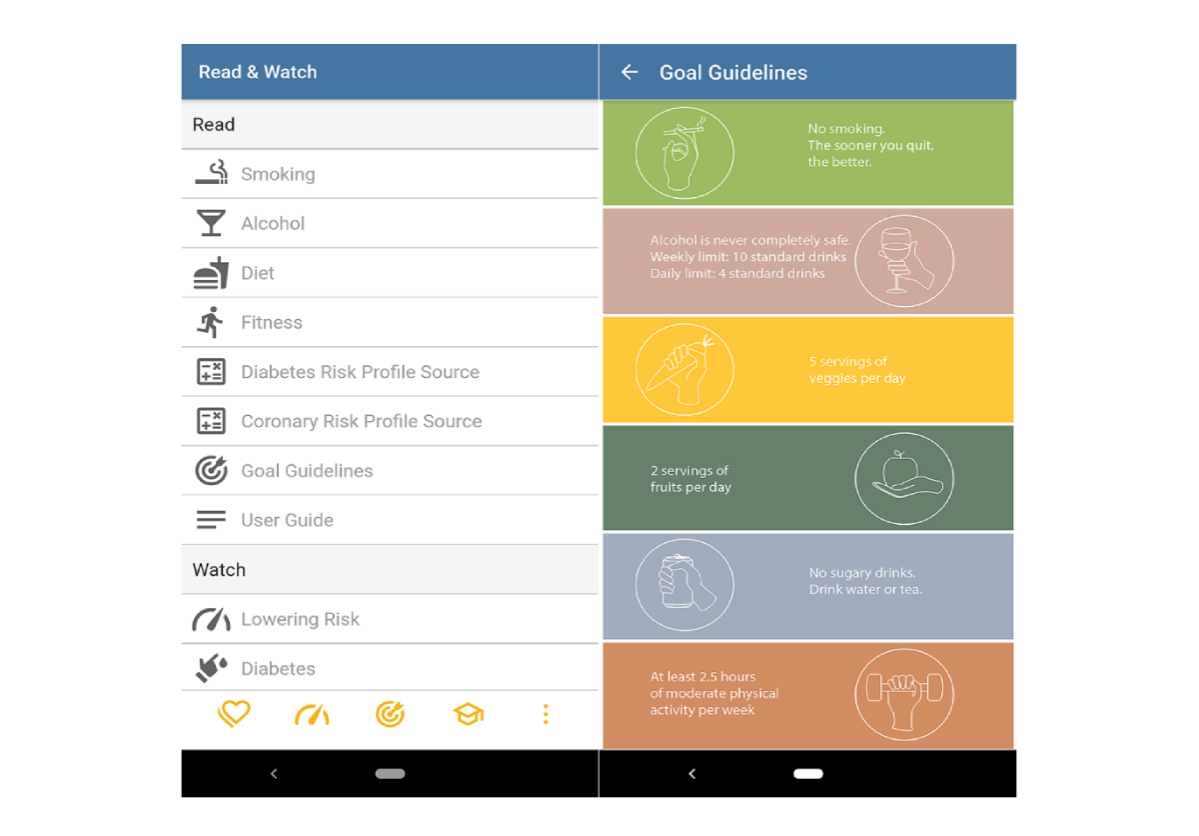
Screenshots of the educational module.

The educational module (Figure 6) contained 5 videos explaining how different risk factors could increase an individual’s risk of developing CVD and T2DM, the difference between modifiable and nonmodifiable risk factors, and behavioral and biomedical risk factors. Each video played for approximately 1 minute, was in plain language, and included captions. Furthermore, for each behavior (smoking, alcohol, diet, and physical activity), there were links to external websites. These websites were Australian and contained evidence-based information about healthy lifestyle. The information was provided to help users to formulate strategies to attain their goals, for example, quit lines for smoking cessation, recipes for a healthy diet, and exercise instructions for a home workout. An infographic (Figure 6) provided a quick overview of the guideline recommendations. This module also included the user guide and a short video that introduced the app.

### Usability Testing Results

We conducted the usability tests between May 2021 and July 2021. In the second round of usability testing, we did not identify any major issues. Hence, we concluded the testing after the second round. A total of 12 individuals provided consent to participate in the study. Of these 12 individuals, 2 (17%) individuals were lost to follow-up and were not included in the analysis. The participants who completed the usability testing were, on average, aged 58 (SD 8) years, ranging from 47 to 67 years. Of the 10 participants, 2 (20%) participants identified as men and 8 (80%) identified as women. Of the 10 participants, 6 (60%) participants owned an iOS phone and 4 (40%) owned an Android phone. When asked about their app experience on a scale of 1 to 5 (1=very unexperienced and 5=very experienced), the average response was 4 (SD 0.9). In the first round, we identified a technical issue that prevented 30% (3/10) of the participants from completing the registration process. Table 2 presents the results of the System Usability Scale. Totally, 71% (5/7) of the participants who did not encounter the issue rated the usability of the app above average (ie, score >68 [64]). The median response for ease of use was strong agreement. The median response for confidence in using the app, frequent app use, and quickly learning to use the app was agreement. The median response for inconsistencies in the app, cumbersome use, and the need to learn much before using the app was disagreement. The median response for the app being unnecessarily complex and requiring technical support for its use was strong disagreement. There were mixed results regarding how well the various functions in the app were integrated (median response was neutrality).

None of the participants faced issues while installing the app. All of them stated that the process was easy and quick. Those who did not experience the technical issue during the registration process said that the process was easy and quick. Participants said that it took them from <1 minute to couple of minutes to download the app and <5 minutes to register. During the interviews, the following issues regarding functionality were identified. A participant mentioned the need to adjust the font size for people with impaired vision. This functionality was already available, but we added an explanation to the user guide about how to adjust the font size through the phone settings. Another participant considered it “self-defeating” to have an extra file for the user guide. Hence, we integrated the user guide into the educational module of the app. In response to that participant’s comments, we included a short video in the educational module that explained the app’s features and their purposes. Regarding language and clarity, we changed some icons and wording that the participants found unintuitive. For example, we changed the tick symbol to a return arrow and used the wording “tap to register” instead of “register.” Another finding regarding language and clarity was the ambiguity about the intended use of the app. A participant wanted the information directly in the app instead of the extra user guide. Another participant explained that it was not clear how often they were supposed to enter their health measures. The participants in the user testing were only asked to download the app and complete the registration process and not to use it for a long period. Therefore, we had not provided them specific information about the frequency of use. For future users, we elaborated on the intended use of the app in the user guide and highlighted that we recommended regular use in the short introductory video.

**Table 2:** Results of the System Usability Scale (n=7).

Statement	Strongly agree, n (%)	Agree, n (%)	Neutral, n (%)	Disagree, n (%)	Strongly disagree, n (%)
I think that I would like to use this app frequently.	0 (0)	5 (71)	1 (14)	1 (14)	0 (0)
I found the app unnecessarily complex.	0 (0)	1 (14)	1 (14)	0 (0)	5 (71)
I thought the app was easy to use.	4 (57)	1 (14)	1 (14)	1 (14)	0 (0)
I think that I would need the support of a technical person to be able to use this app.	0 (0)	1 (14)	0 (0)	1 (14)	5 (71)
I found the various functions in this app were well integrated.	1 (14)	2 (29)	1 (14)	3 (43)	0 (0)
I thought there was too much inconsistency in this app.	0 (0)	1 (14)	1 (14)	3 (43)	2 (29)
I would imagine that most people would learn to use this app very quickly.	1 (14)	4 (57)	1 (14)	1 (14)	0 (0)
I found the app very cumbersome to use.	0 (0)	1 (14)	1 (14)	2 (29)	3 (43)
I felt very confident using the app.	3 (43)	3 (43)	0 (0)	1 (14)	0 (0)
I needed to learn a lot of things before I could get going with this app.	0 (0)	0 (0)	1 (14)	3 (43)	3 (43)

Regarding the content, a few participants expressed interest in the risk scores that were being calculated in the app. A participant stated the following:

And yet, it showed up that I was at moderate risk of getting diabetes in the next five years, and I thought: ‘What on Earth is that based on?’ and it undermined my confidence in the app.

P10

To show that the risk scores were from credible sources, we included links to the websites for the risk scores in the educational module. Furthermore, some participants wanted to see time trends for the health measure. In response to their comments, we included a graphical display for each health measure that showed the progress over time. A participant expressed confusion about the external websites that we linked to in the education section. Another participant suggested a summary of the most important information about the health behaviors in the app in a visually appealing form. In response to these comments, we added some information about the external websites to the user guide and instructional video. In addition, we added an infographic to the educational module that summarized the guideline recommendations regarding the 4 behaviors. A few participants commented on the blood pressure and lipid levels that were required for the registration. Comments from the participants include the following:

I couldn‘t answer my cholesterol levels. I couldn’t remember them.”

P6

Why are they only asking about HDL? And why aren’t they asking about LDL?

P10

Another participant explained the following regarding the registration process:

...my only thoughts when I was doing it that some people, uhm, wouldn’t know what a systolic or a diastolic pressure was and so that may be something that you may need. A little explanation of what that is.

P7

We had already anticipated that users might not know their blood pressure or cholesterol levels. Hence, we included an option to use average values instead for the registration. The interviews further highlighted an issue that led us to add 2 educational videos explaining the relationship between disease risk, blood pressure, and cholesterol.

During the interviews, 3 of the participants mentioned commercial apps. Totally, 1 of them commented positively on the risk score module, but criticized that commercial apps would be better at incentivizing the user to continue by showing them how changes in their behavior influenced their risk and by providing incentives when the user achieved their target behavior. The remaining 2 participants said that many commercial apps automatically tracked many daily features, and concluded that this might not be required for our app. Comments from the participants include the following:

There’s lots of different apps in the market, isn’t there, to collect health data like that on a daily basis and monitor it. I don’t know if you need to go down that track of providing a trend, given that there’s so many other competitors in the market.

P10

You know some of the fitness apps or whatever that I’ve been on they have actually almost too, too much stuff. I mean, I think this app is, is very good for kind of, you know, kind of like the basics.

P9

Similarly, another participant explained the following:

I don’t know what your demographic for the app is, but my parents are in their 90s and I think at least one of them would be able to, to use it, with a bit of help.

P1

## Discussion

### Principal Findings

We developed an app that will form part of an intervention for the prevention of CVD and T2DM. The app’s role in the intervention will be to make users aware of their disease risk and to engage them in healthy behavior. We developed the app around the principles of BCW to achieve a robust app construct. In total, we incorporated 12 behavior change techniques into the app to increase the capability, opportunity, and motivation of users to change their behavior. During the usability testing, participants ranked the usability of the app above average, based on the System Usability Scale. They stated that the app was easy and quick to download, basic in design, and easy to use. We used the participants’ feedback to eliminate technical errors and adapt the app to their wishes and needs. Regarding the intervention, we do not anticipate that every user will adopt the *ideal* behaviors as described by the guidelines. For example, we do not expect that simply by using the app, a heavy drinker will stop consuming alcohol, a person who is obese will achieve normal weight, or a smoker will quit smoking. However, even small changes in behavior can decrease an individual’s risk for CVD and T2DM. In addition, we do not consider the app as a stand-alone tool. Instead, we anticipate that app users will learn about their disease risk, risk behavior, and the connection between the 2 and that the app will help them seek information about where to receive help if needed (eg, from their GP or through support programs).

### Comparison With Previous Studies

A similar study from Singapore that targeted coronary heart disease prevention via an app measured risk awareness, knowledge of risk factors, perceived stress levels, and heart-related lifestyle measures as outcomes [91]. Jiang et al [91] concluded that the intervention increased risk awareness and disease knowledge and the effects persisted for at least 6 months. They did not measure disease risk or incidence as outcomes [91]. The intervention of Jiang et al [91] differed from ours, as it focused on a 28-day time frame in which participants additionally received daily SMS text messages. In addition, the app included a stress management module, and the focus of the app was on written educational content including short quizzes [91,92]. The app features that we implemented were more diverse, including goal setting and tracking of different behavioral risk factors. Redfern et al [19] recruited Australians who are at moderate to high risk of CVD via GPs for their digital health intervention for CVD prevention. Similarities between their intervention and ours were that participants could learn about the relationship between CVD risk and their lifestyle, were encouraged to talk to their GP about these topics, and could set goals and monitor their lifestyle behavior [19]. Differences were that their intervention also focused on medication adherence and included data input from the electronic health record [19]. Redfern et al [19] concluded that the intervention was not successful at improving medication adherence, which was the primary outcome.

Plotnikoff et al [93] developed an app-based intervention for T2DM prevention in Australia. In contrast to ours, a noteworthy proportion of their intervention was delivered in person in the form of cognitive training and exercise classes [93]. This limits the ability of the intervention to be scaled up. Block et al [14] developed a fully automated digital intervention for T2DM prevention in people who are prediabetic. The program consisted of weekly tailored goal setting and tracking of behaviors for the first 6 months and fortnightly for the following 6 months [14]. The intervention led to improved diabetes biomarkers and overall decreased T2DM risk [14]. In addition, it positively affected diet-related and physical activity–related behaviors [94]. The intervention differed from ours in that it included behavioral support for stress and sleep in addition to physical activity, diet, and weight loss [14]. It also comprised a website, automated phone calls, and emails [14]. Another difference was that it included social comparison features such as team competitions [14]. The theoretical base for competitions is the social upward comparison [95]. According to Spohrer et al [95], the social upward comparison theory is not compatible with the protection motivation theory because, in combination, they would lead to negative effects. We focused on aspects of the protection motivation theory. More specifically, the risk assessment module targets threat appraisal and the other modules target coping appraisal.

### Implications and Future Research

We designed the app as simple as possible, so that it could be a tool for laypeople to use on their own. Ideally, it should encourage users to recognize their risks and make lifestyle-related changes without the direct need for medical or technical support. However, if they are at high risk of developing CVD or T2DM, engaging with the app should alert users and encourage them to seek help from their GP. We believe this is what sets our study apart from previous studies, which has tended to focus on people at high risk, includes the involvement of health care providers, or both. A recent systematic review of mobile health apps for the management of chronic conditions by Cucciniello et al [96] showed that the studies with additional human-led components did not have higher likelihood of positive effects on the outcomes for those in the intervention group. However, we believe that a few points should be considered when there is no direct involvement of health care professionals in the intervention. These include the appropriate promotion and uptake of the intervention to ensure that those who are likely to benefit are aware of and have access to it. In addition, the intervention should be designed such that the users who are at high risk will use it with medical supervision. Currently, we are in the process of evaluating the feasibility of this app-based intervention. Depending on the results of the feasibility study, we intend to conduct a study to evaluate the effectiveness of the app.

### Strength and Limitations

A strength of our app was that the development process was guided by scientific evidence, with a focus on the APEASE criteria. We provided a detailed description of the theoretical principles and design considerations, which showed transparency as opposed to the *It Seemed Like A Good Idea At The Time* principle [57]. This enabled the research team to understand which app features might be effective in changing the user’s behavior. It also allows other researchers to replicate this study. Another strength was that we included feedback from potential users in the development process. A limitation was that the intervention focused on changes that were needed in the person rather than in the environment. The BCW from which we built the theoretical base comprised 9 intervention functions. We did not address 4 functions, such as coercion, restriction, environmental restructuring, and modeling, which, according to Michie et al [32], focus on external influences. Our app-based intervention focused on the personal agency of the participants. We limited diet-related risk factors to vegetables, fruits, and sugary drinks, which could potentially undermine the importance of other diet aspects, such as salt and whole grain intake. However, this was a conscious choice based on previous studies, which suggested that vegetables, fruits, and sugary drinks were easier to track than other diet-related behaviors. Another limitation was that the app relies on user input. It does not automatically collect information, for example, step count or data from the electronic health record. We made these choices owing to reliability and privacy issues.

### Conclusions

This paper describes the theoretical framework, design process, and usability testing of an app that will form the basis of an intervention for the primary prevention of CVD and T2DM. The app addressed the 3 behavior components, capability, opportunity, and motivation, which are core components of the BCW. In the usability testing, the participants rated the apps’ usability as above average, according to the System Usability Scale. Most participants found the app easy to use, and they thought that most people would learn to use the app quickly. They also showed interest in using it frequently. After the user testing, some additional functions requested by the participants were integrated into the app. For example, a short introductory video and graphs that show the health measures over time were included. Next, we will use the revised version of the app that resulted from this design process and usability testing in a feasibility study.

## Appendix

Multimedia Appendix 1 Additional information on the 4 behavioral risk factors, design principles, and evidence for the effectiveness of certain behavior change techniques.

Multimedia Appendix 2 Android version of the user guide.

Multimedia Appendix 3 Interview guide.

## Abbreviations

APEASE: affordability, practicability, effectiveness and cost-effectiveness, acceptability, side effects and safety, and equity
BCW: Behaviour Change Wheel
COM-B: capability, opportunity, and motivation for behavior change
CVD: cardiovascular disease
GP: general practitioner
iOS: iPhone Operating System
T2DM: type 2 diabetes mellitus
