# Supplementary material for: A Mobile App for Prevention of Cardiovascular Disease and Type 2 Diabetes Mellitus: Development and Usability Study
Source: JMIR Hum Factors. 2022 May 10;9(2):e35065. doi: 10.2196/35065 (PMC9131155; doi:10.2196/35065)
Supplement: Multimedia Appendix 1 [file humanfactors_v9i2e35065_app1.pdf]

This is a Multimedia Appendix to a full manuscript published in JMIR Human Factors. For full copyright and citation information see http://dx.doi.org/10.2196/35065

Table S1. Percent of the population aged 45 years and older (by sex) who perform the 4 risk behaviors [46], and percent of linked disease burden (expressed as DALY) [45].

Behavior	Definition of risk	Risk prevalence (%)		Attribution to CHD[a] burden (%-DALY[b])		Attribution to T2DM[c] burden (%-DALY)	
		Men	Women	Men	Women	Men	Women
Tobacco use	Daily cigarette smoking	14.2	11.1	15.8	10.4	6.5	1.6
Alcohol use - lifetime risk	> 2 standard drinks on any day	26.4	9.2	2.5[d]	5.4[d]	—	—
Alcohol use - single occasion risk	> 4 standard drinks on single occasion	44.8	20.6	—	—	—	—
Diet low in fruit	< 2 serves per day (excluding juices)	48.8	38.9	8.7	7.2	7.6	7.3
Diet low in vegetables	< 5 serves per day (excluding juices)	95.0	87.2	14.3	12.8	—	—
Diet high in sugary-sweetened drinks	Daily consumption	9.4	5.2	0.7	0.3	0.9	0.6
Physical inactivity	Moderate to vigorous activity per week[e]	83.8	85.4	12.0	11.3	18.5	20.1

[a]CHD, coronary heart disease.
[b]DALY, disability-adjusted life years.
[c]T2DM, type 2 diabetes mellitus.
[d]Definition of exposure: average daily alcohol consumption by current and former drinkers.
[e]For 45-64-year-olds < 150 min, for 65+-year-olds < 30 min[d], vigorous activity multiplied by 2.

Table S2: Overview of studies reporting on behavior change techniques with positive effects in digital intervention for lifestyle changes.

First author, year, reference	Study type	Target behavior or outcome, target group	Behavior change techniques with positive effects
Carraça, 2021, [21]	Systematic review and meta-analysis	Physical activity, overweight or obese adults	Goal setting behavior; goal setting outcome; graded tasks; social incentive
Schroé, 2020, [54]	Factorial randomized trial	Physical activity	Coping planning; self-monitoring
Qin, 2021, [24]	Systematic review and meta-analysis	Physical activity for weight loss	Feedback on behavior
Asbjørnsen, 2019, [55]	Scoping review	Long-term weight loss and maintenance, overweight or obese adults	Self-monitoring; feedback; goal setting; shaping knowledge; social support
Van Rhoon, 2020, [47]	Systematic review	T2DM[a] prevention	social support (unspecified); goal setting behavior; goal setting outcome; feedback on behavior; self-monitoring of outcomes of behavior; self-monitoring of behavior; and problem solving
Kaner, 2017, [23]	Systematic review and meta-analysis	Alcohol intake, people living in community	Behavior substitution; problem solving; credible source
Garnett, 2015, [48]	Formal consensus	Alcohol intake	Self-monitoring; goal setting; action planning; feedback in relation to goals
McCrabb, 2019, [49]	Systematic review and meta-analysis	Tobacco smoking	Short-term: goal setting behavior; problem solving; action planning; pros and cons; pharmacological support; Long-term: problem solving; action planning; social support unspecified; natural consequences; pros and cons; pharmacological support
Black, 2020, [25]	Systematic review and meta-analysis	Tobacco smoking	Prompting commitment; social reward; identity

[a]T2DM, type 2 diabetes mellitus.

Table S3: Design principles

I. Design principles adopted from older user accessibility guide [50]
Option to enlarge the font size
Videos include caption
Colors with good contrast
Clear layout and design
Large buttons
Understandable content
Notifications and feedback: easy-understandable messages
II. Additional design principles adopted from previous research
Privacy and security [51-53]
Ensure within-app consistency [53]
Simplify within-app navigation [53]
Streamline data entry process [53]
Enhance and simplify data visualizations [53]
Offline access [53]
III. Additional design principles identified by the research team
Low internet data usage
Small storage space on the phone
Available for iOS and Android phones
