# Supplementary material for: A Mobile App for Prevention of Cardiovascular Disease and Type 2 Diabetes Mellitus: Development and Usability Study
Source: JMIR Hum Factors. 2022 May 10;9(2):e35065. doi: 10.2196/35065 (PMC9131155; doi:10.2196/35065)
Supplement: Multimedia Appendix 2 [file humanfactors_v9i2e35065_app2.pdf]

# User Manual

# Risk Track App

Australian e-Health Research Centre
& University of New South Wales
User Manual

# Risk Track App

## User Manual

**Author: Vera Buss**

**Version 3.1 – Android**

Copyright: Australian e-Health Research Centre and University of New South Wales

# Contents

Introduction....3

Important Points....3

Installing the App....4

Registration....5

Setting Goals....7

Important Points....7

Risk Score....8

Health Measures....9

Read & Watch....11

Frequently Asked Questions....12

Privacy Notice....12

# Introduction

The Risk Track App is designed to help you understand your risk of developing cardiovascular disease and diabetes. You can set health goals and track your progress towards these. If you stick to healthy habits, this may lower your risk for cardiovascular disease and diabetes. This manual will provide a step-by-step guide to using the Risk Track App.

## Important Points

- The Risk Track App can help you understand your risk if you do not have cardiovascular disease or diabetes. The app is not intended to help people self-manage these diseases.
- You should talk to your GP or another health professional if the Risk Track App displays a high risk for you or if you have any other concerns.
- You need to be aware that you need to adopt a healthy lifestyle in the long term to decrease your risk of cardiovascular disease and diabetes.

# Installing the App

1. Go to the Play Store
2. Search 'AEHRC'
3. Select the Risk Track App
4. Select 'OPEN'
5. Select 'INSTALL'
6. Once installation completed, select 'OPEN'

7. Enter your unique identifier

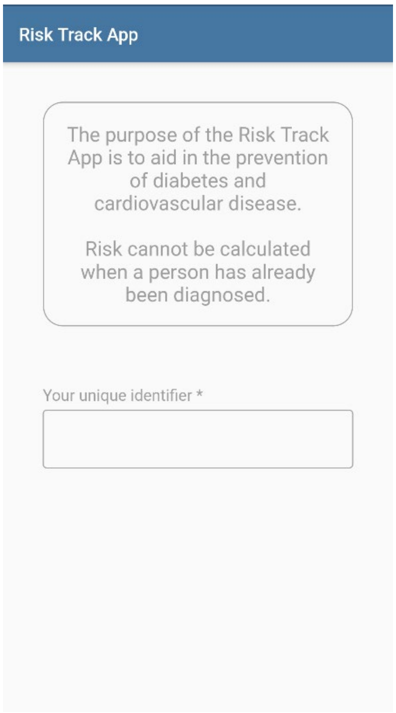


8. Click 'Tap to Register'

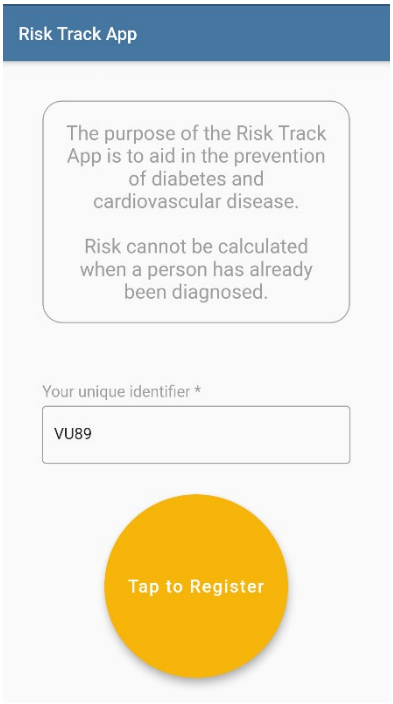

# Registration

1. Fill out demographic information

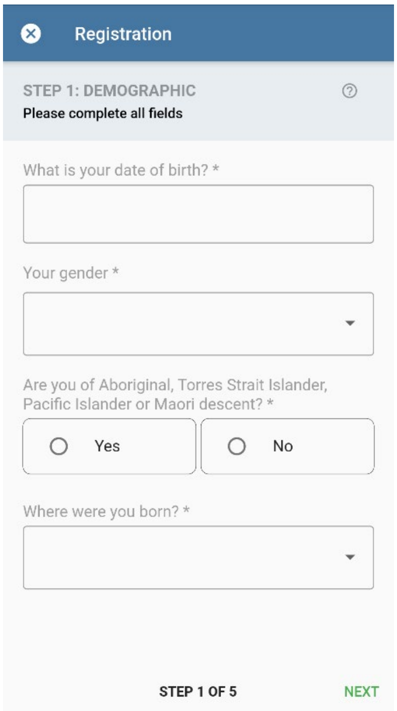


a. Select date of birth using the calendar

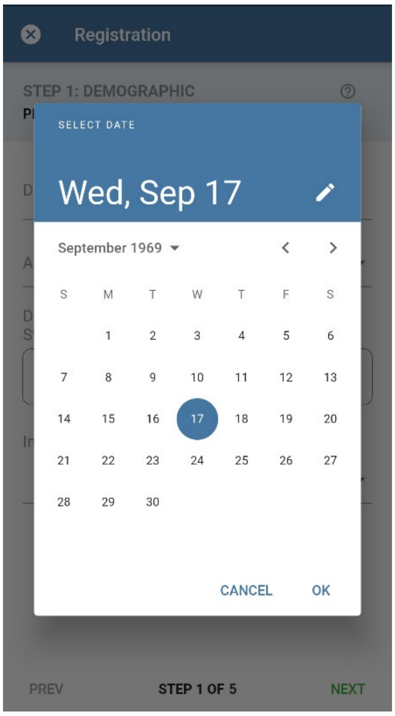


b. Click ‘NEXT’ to continue

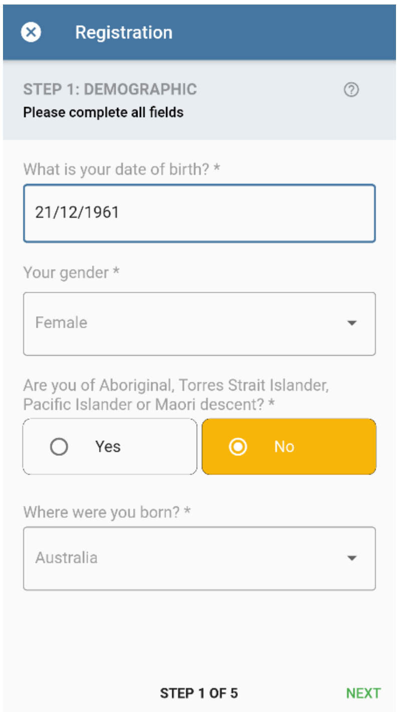


2. Fill out Medical History I,

then click ‘NEXT’

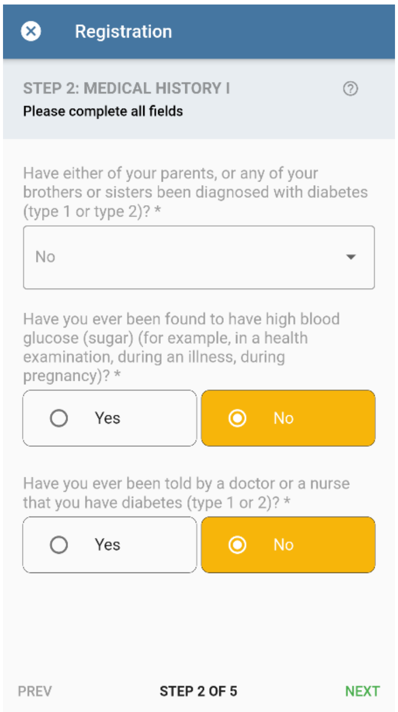

3. Fill out Medical History II,

then click 'NEXT'

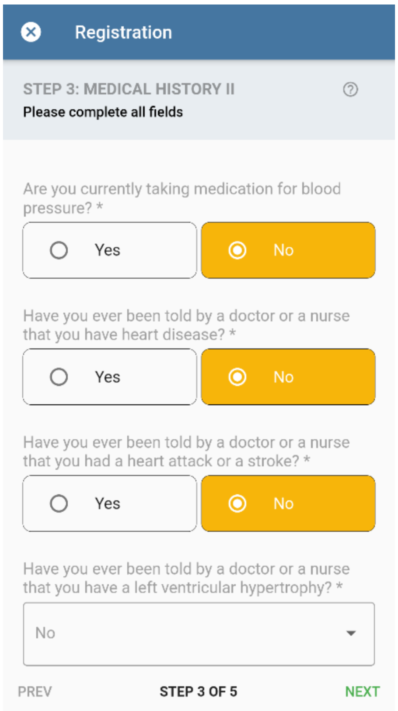


4. Fill out Lifestyle information,

then click 'NEXT'

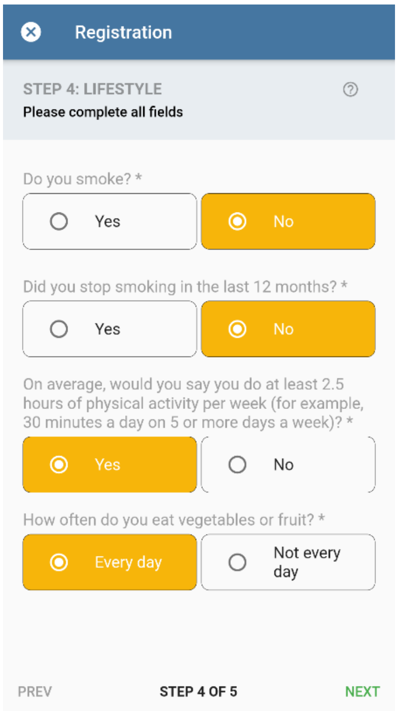


5. Fill out the information about Your

Health Today, then click 'NEXT'

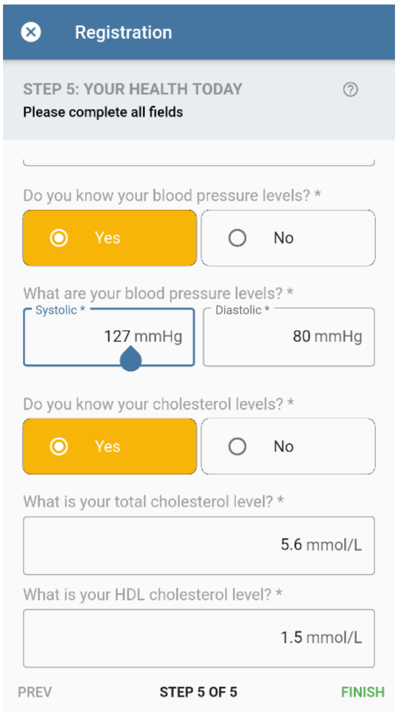


Your registration is complete.

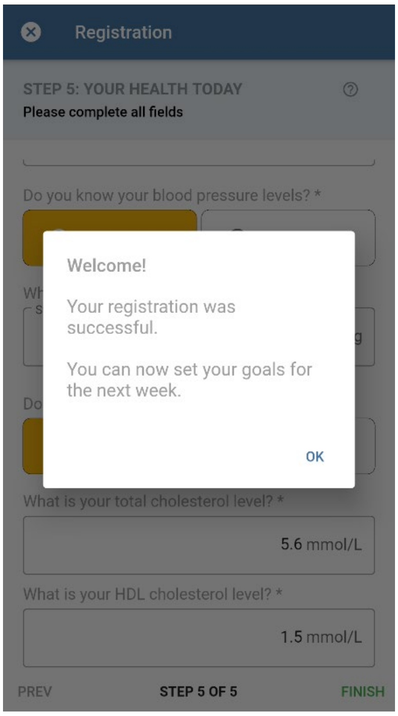

# Setting Goals

Please start by setting for each Health Measure a goal that you know you can do. Once you have achieved the goal, you can aim for something more ambitious. As a guideline, we provide values from which you can select your goal. These are based on the Australian guidelines.

## Important Points

- *Your Health Measures are specific to your goals and allow you to follow up on your progress towards these.*
- *Physical Activity and Alcoholic Drinks are measured per week.*
- *For Smoking: It depends on your smoking habits if you measure smoke-free days per week or cigarettes per day (which also includes other tobacco products). If you selected that you do not smoke during the registration process, smoking will not be displayed.*
- *All other Health Measures are counted per day.*
- *For Physical Activity: If you did any exercise which caused a large increase in your heart rate or breathing, that is, vigorous exercise, please multiple this time by 2. If you walked consecutively for 10 minutes or longer, add this time to your Physical Activity.*
- *For Sugary Drinks and Alcoholic Drinks: If you select that you do not drink these, they will not appear in the Health Measures.*

Select for each Health Measure a value from the list as your goal.

If you want to update or review your goal later, just select the -button.

You can then see for each Health Measure the value you have last achieved.

You can find more information about guideline recommendations for your goals in the Read & Watch section.

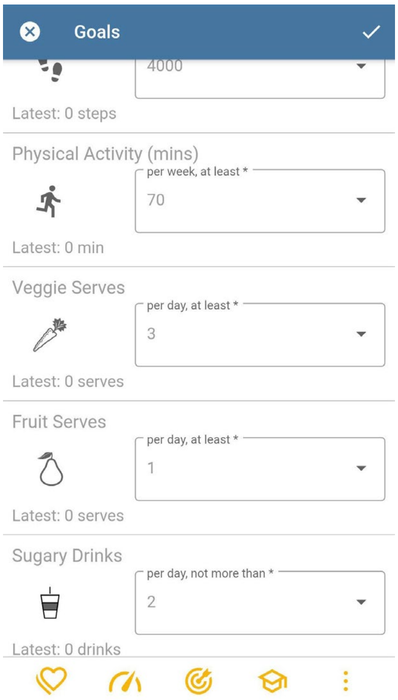


# Risk Score

## Here you see your current risk for developing diabetes and cardiovascular disease in the next 5 years displayed.

You can update your risk information at any time.

Click on the -icon to provide updated information.

Some of the information that is required to calculate your risk score is captured through the Health Measures.

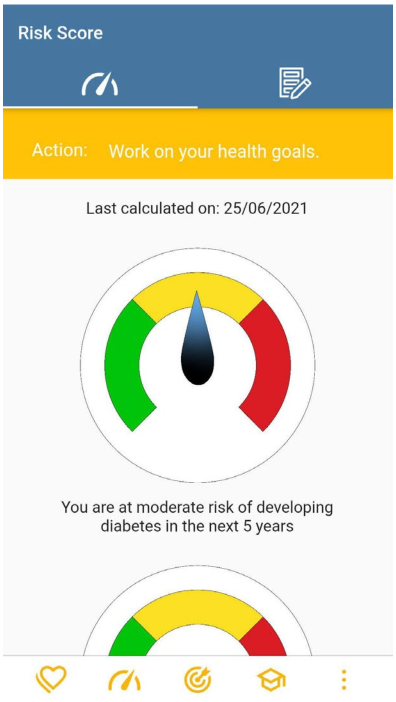

# Health Measures

Here you can track your daily progress towards your goals. It is up to you how often you enter values. The more frequent you enter your health measures, the better you can understand your behaviour.

Click on the -icon to get to the Health Measures.

There are two options to provide information:

1. Click briefly on the number of the Health Measure type to enter a value.

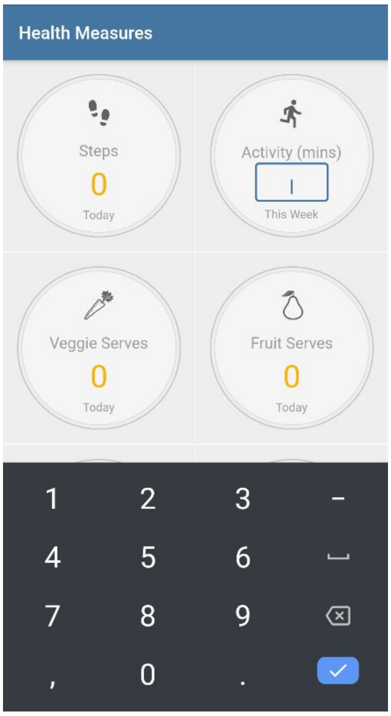


2. Click longer on the number to add one to the previous value.

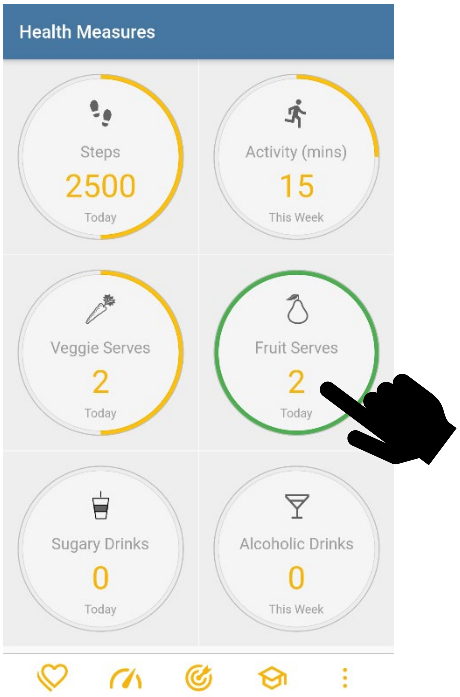


You can view your progress towards a goal by completeness of the corresponding circle. For example, for steps, your goal has been met when the circle is fully green, while for sugary drinks the circle turns red once you have had more sugary drinks than you set as your maximum for the day.

If you click directly on the Health Measures icon, you can see your progress over time.

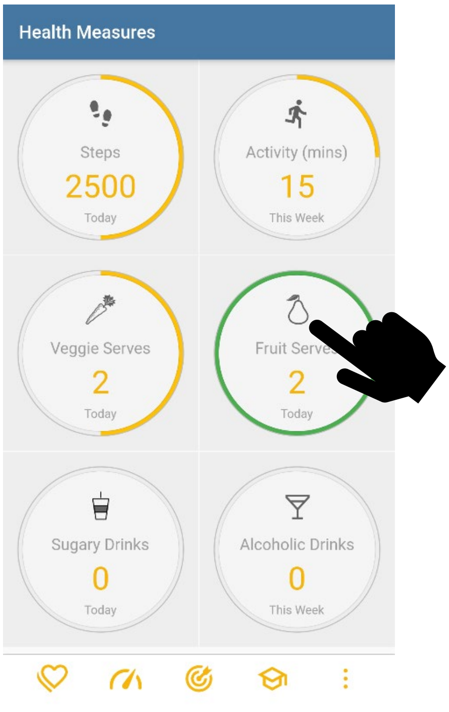


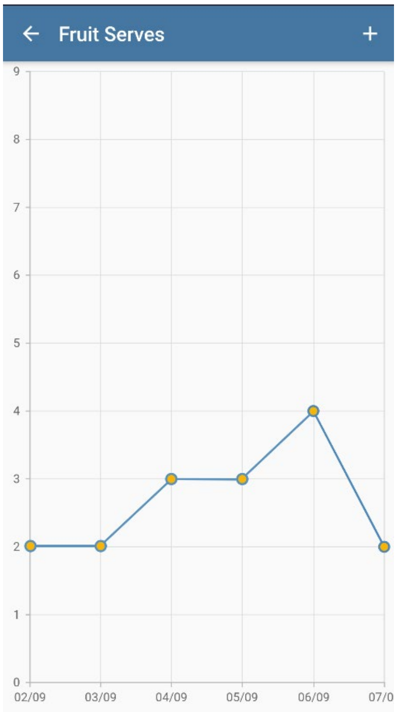


You can also add or change previous data entries.

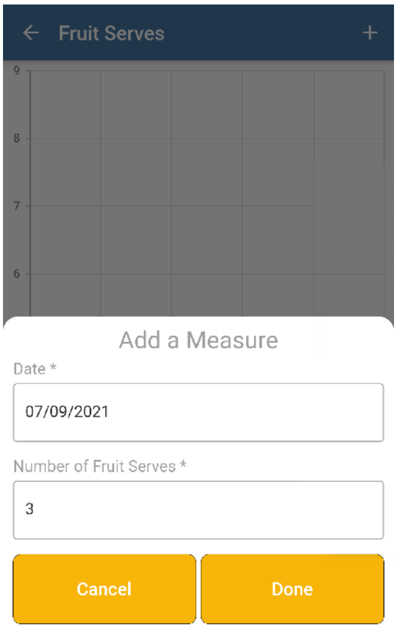

# Read & Watch

Here you can access information regarding cardiovascular disease, diabetes, and how to lower your risk.

Select the -icon to get to the educational content. Select the topic you wish to view:

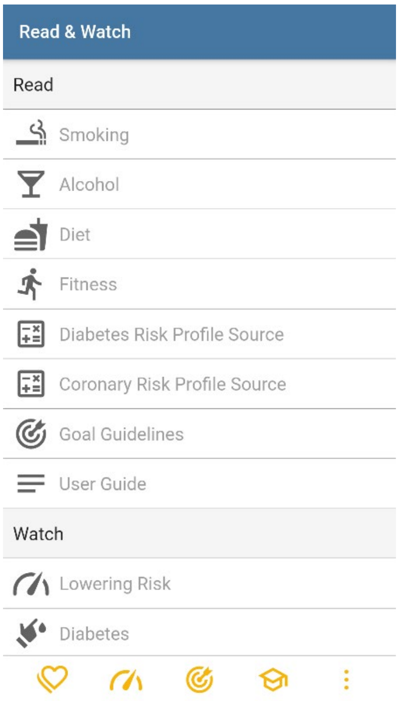


These lead to external websites. The first four websites include information about a healthy lifestyle. These websites are from Australian governmental institutes or charities. You can browse them to learn more about the health recommendations. They can also help you to develop strategies to achieve your health goals. The two below lead you to the original risk calculators.

These are five short educational videos. There is also a quick video that gives you an introduction into the app.

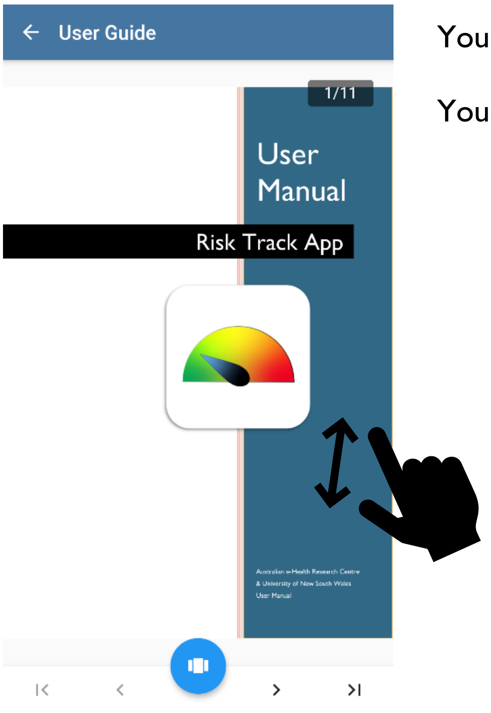


You can read the User Guide through the app.

You can zoom in using your fingers.

# Frequently Asked Questions

***Is there a cost to download the app?*** – The app is free to use.

***Will the app use a lot of data?*** – Total data usage is low. Total data usage is estimated to be equivalent to viewing a few images on the internet or Facebook, assuming the app is downloaded once and used as recommended.

***Which smartphones does the app run on?*** – As a guide, if the Risk Track App shows up in the Google Play store, it should run on your smartphone.

***Will I be taught how to use the app?*** – This user manual is intended to provide you with all the information required to use the app.

***Can I change my mind?*** – You are welcome to opt-out of the study at any time.

***How can I change the display size?*** – Open your phone's Setting app . Select Accessibility . Go to 'Display size'. Set your preferred display size with the slider.

---

# Privacy Notice

The information you provide is personal information for the purposes of the Privacy and Personal Information Protection Act 1998 (NSW). You have the right of access to personal information held about you by the University, the right to request correction and amendment of it, and the right to make a complaint about a breach of the Information Protection Principles as contained in the PPIP Act. Further information on how the University protects personal information is available in the UNSW Privacy Management Plan.
