# Supplementary material for: A Mobile App for Prevention of Cardiovascular Disease and Type 2 Diabetes Mellitus: Development and Usability Study
Source: JMIR Hum Factors. 2022 May 10;9(2):e35065. doi: 10.2196/35065 (PMC9131155; doi:10.2196/35065)
Supplement: Multimedia Appendix 3 [file humanfactors_v9i2e35065_app3.pdf]

Interview Guide

Introduction

Hi [name]. My name is [researcher name] from the University of New South Wales. I am contacting you as arranged by [contact person] to participate in our study about a smartphone application. I wanted to give you a little information about what you will be looking at and give you time to ask any questions you might have before we get started.

Description of the study

Today we are asking you to serve as an evaluator of a smartphone application. Our goal is to see how easy or difficult you find the smartphone application. From now onwards, I will just say ‘app’ instead of smartphone application to keep it simpler.

Invitation to participate and have interview recorded

I would like to confirm and reassure you that all comments and opinions that arise during this interview will remain confidential. You are free to withdraw your consent or discontinue participation at any time without prejudice.

The interview will be audio-recorded and transcribed, and you will be provided with a pseudonym to protect your privacy. The interview transcription will be used only for the purpose of coding interview data regarding this project. Reports or manuscripts will be written using the findings from this discussion, but the results will be presented as themes, and if we refer to your comments, we will use a pseudonym.

Do you have any questions?

Confirmation of consent:

Prior to this meeting, you have already provided us with your consent to participate in this research. Can I please confirm you give your consent?

☐ No → **[Confirm withdrawal from study.]**

> Can you please confirm that you withdraw you consent, and you would like any identifiable information collected about you which you have provided for the purpose of this research study withdrawn.
>
> ☐ No → **Do you have any questions?**
> ☐ Yes → **Thank you for your time.**

☐ Yes → **Continue.**

Procedure

Before this interview, you received instructions on how to download and navigate through the app. During this session, I would like you to tell me what your thoughts and experiences were while you worked to complete these tasks. From time to time, I may ask you to clarify what you have said or ask you for information on what you were looking for or what you expected to have happened. There will also be a few questions for you to answer at the end of the exercise.

Do you have any questions before we begin?

[Answer questions as appropriate]

1. ***Downloading the app***

I provided you beforehand with a leaflet to explain how to download the app on your phone.

Were you able to download the app?

☐ **No**

- → What was the problem?
- → At which stage did you get stuck?
- → Did you look at the provided User Guide?
- → Do you have any suggestions how the downloading process could be easier?

- → Proceed to ***4. Demographic information***

☐ **Yes**

- → Did you have any questions or difficulties that you encountered?
- → How long did it roughly take you to download the app?
- → Did you look at the provided User Guide?
- → Do you have any suggestions how the downloading process could be easier?

2. ***Using the app***

Were you able to create a user profile?

☐ **No**

- → What was the problem?
- → At which stage did you get stuck?
- → Did you look at the provided User Guide?
- → Do you have any suggestions how the process could be easier?

- → Proceed to ***4. Demographic information***

☐ **Yes**

- → Did you have any questions or difficulties that you encountered?
- → How long did it roughly take you to create the user profile?
- → Did you look at the provided User Guide?
- → Can you tell me the likelihood of developing heart disease and diabetes that the app showed you based on the information of the dummy profile?
- → Do you have any suggestions how the registration process could be easier?

3. Assessing the app

I will now read to you 10 statements. Please rate how much you agree with these statements on a scale from 1 to 5, where 1 means you strongly disagree and 5 that you strongly agree with the statement.

Table 1: System Usability Scale

	Strongly disagree<br>1	2	3	4	Strongly agree<br>5
1. I think that I would like to use this system frequently.	☐	☐	☐	☐	☐
2. I found the system unnecessarily complex.	☐	☐	☐	☐	☐
3. I thought the system was easy to use.	☐	☐	☐	☐	☐
4. I think that I would need the support of a technical person to be able to use this system.	☐	☐	☐	☐	☐
5. I found the various functions in this system were well integrated.	☐	☐	☐	☐	☐
6. I thought there was too much inconsistency in this system.	☐	☐	☐	☐	☐
7. I would imagine that most people would learn to use this system very quickly.	☐	☐	☐	☐	☐
8. I found the system very cumbersome to use.	☐	☐	☐	☐	☐
9. I felt very confident using the system.	☐	☐	☐	☐	☐
10. I needed to learn a lot of things before I could get going with this system.	☐	☐	☐	☐	☐

© Digital Equipment Corporation, 1986.

Acknowledgement: System Usability Scale was developed as part of the usability engineering programme in integrated office systems development at Digital Equipment Co Ltd., Reading, United Kingdom.

4. Demographic information

I just have three more questions for you. Could you please tell me now your age and gender?

Age: __________________

Gender: ________________

How would you rate your experience with smartphone apps on a scale from 1 to 5, where 1 means you are very unexperienced and 5 very experienced?

Very unexperienced				Very experienced
1	2	3	4	5
☐	☐	☐	☐	☐

Do you have any further comments or suggestions about the app or our instructions?

Conclusion

As promised, we will provide you with a $20 Coles/Myer gift card to thank you for your participation. We will send it to the address you provided us with on the consent form.

Thank you for participating in this usability testing, your help is greatly appreciated. We really appreciate your answers and if you think of any additional comments or questions, please feel free to send us an email to the address in the Participant Information Sheet

Resource used to create this guide:

U.S. Department of Health & Human Services (2020). Introduction to Testing with Moderator Interaction. Usability.gov. Accessed: 06 March 2020. https://www.usability.gov/how-to-and-tools/resources/templates/introduction-to-testing-with-moderator-interaction.html
